# A Rare Cause of Intranasal Mass: Bilateral Ectopic Nasal Teeth

**Published:** 2017-09

**Authors:** Hasan-Emre Koçak, Kadir Özdamar, Bekir Bilgi, Harun Acıpayam

**Affiliations:** 1 *Department* *of* *Otolaryngology Head and Neck Surgery,* *Bakırköy Dr.Sadi Konuk Training and Research Hospital, Istanbul, Turkey.*; 2 *Department* *of* *Otolaryngology, Suruc State Hospital, Sanliurfa, Turkey.*

**Keywords:** Bilateral, Intranasal, Obstruction, Tooth

## Abstract

**Introduction:**

Ectopic teeth occur in a wide variety of sites, including the maxillary sinus, mandibular condyle, coronoid process, orbital, and nasal cavities. Reported symptoms and signs associated with nasal teeth include facial pain, external nasal deformities, foul-smelling rhinorrhea, recurrent epistaxis, and oronasal fistula. Ectopic teeth occurring bilaterally in the nasal cavity is very very rare.

**Case Report:**

A bilateral intranasal ectopic teeth case, which is asymptomatic on the right side and symptomatic on the left side, is presented. The tooth on left side was extracted endoscopically. There were no complications.

**Conclusion:**

Extraction of an intranasal tooth under endoscopic guidance is an adequate treatment. If the ectopic intranasal tooth is asymptomatic, clinicians should follow with clinical examination and radiological imaging.

## Introduction

Ectopic teeth are very rare and have a rate of 0.1-1% clinical cases seen in the community (1). They are frequently seen on the palate and maxillary sinus. It can occur in a wide variety of locations including the maxillary sinus, mandibular condyle, coronoid process, orbital, and nasal cavities. Ectopic teeth found bilaterally in the nasal cavity is very rare. Reported symptoms and signs associated with nasal teeth include facial pain, external nasal deformities, foul-smelling rhinorrhea, recurrent epistaxis, and oronasal fistula (2). We present a case of bilateral intranasal ectopic teeth, which is asymptomatic on the right side and symptomatic on the left side.

## Case Report

**Fig 1:**
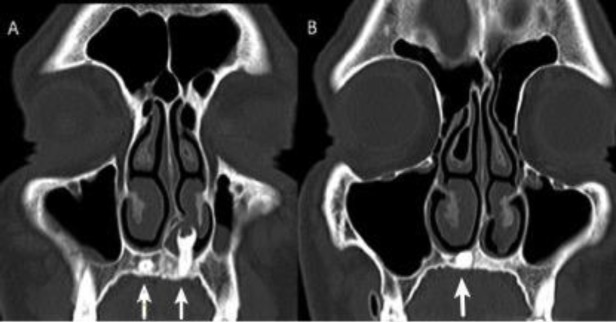
Coronal section of paranasal CT

A 34-year-old man was referred to our clinic. His symptoms were left nasal obstruction, foul-smelling discharge, and occasional nasal bleeding. His symptoms had been lasting for three months. During physical examination, the foul-smelling discharge in the left nasal cavity was aspirated. On the left side of the nasal cavity, a hard, immobile, white colored mass was observed between the deviated nasal septum and nasal sill extending to the inferior surface of the inferior turbinate. The tip of the mass was covered with nasal mucosa. It looked like a tooth. Onn the right side of nasal cavity, we observed mucosal bulging on the nasal sill and right turbinate hypertrophy. The patient was asymptomatic on the right nasal cavity. A coronal section of a paranasal CT scan of the patient was observed and a mass of bone density arising from the left nasal cavity floor was seen (Fig.1). 

A: left ectopic tooth extended vertically in the intranasal cavity

B: Right ectopic tooth extended horizontally in the intranasal cavity.

**Fig 2:**
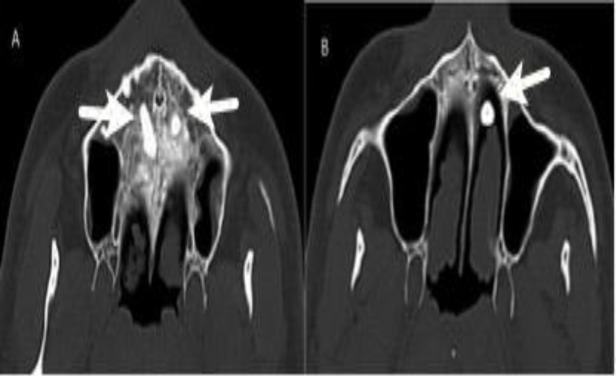
Axial section of paranasal CT

The mass in the left nasal cavity had the same appearance and density of the tooth. This structure was evaluated as an ectopic tooth. In addition, another ectopic tooth was observed in the right nasal cavity. The axial section of the paranasal CT scan of this patient was observed and an ectopic tooth along the base of the nasal cavity was seen (Fig.2). 

A: left ectopic tooth extended vertically in the intranasal cavity

B: Right ectopic tooth extended horizontally in the hard palate

**Fig 3:**
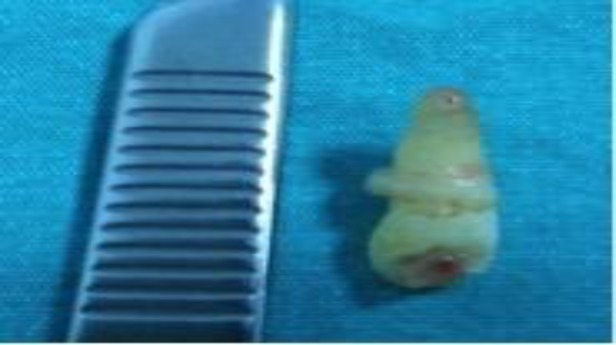
Postoperative photo of the left intranasal ectopic tooth (2cm in length

Extension of the right nasal cavity was minimal. The patient was operated under general anesthesia. After removing the left sided deviation of the cartilage septum, we observed a hard white colored bone, which looked like a tooth and was vertical to the direction of the nasal sill. The left ectopic tooth was excised using punch forceps. It was 2cm in length. The root of the tooth was 1cm in length (Fig.3). 

The tip of tooth was covered with mucosa. Nasal packing was administered in the nasal cavity to prevent bleeding after surgery and was removed after two days. The right ectopic tooth was just followed, because it was asymptomatic. There were no complications (bleeding, synechiae, or repeated complaints). There were no complaints after the operation or during the one-year follow up.

## Discussion

Intranasal ectopic tooth is a very rare clinical condition and symptoms are similar to a clinical intranasal mass. In the literature, unilateral ectopic teeth are more common. Bilateral involvement and multiple teeth in the unilateral nose has also been reported, although rarely. In this report, we present a case of bilateral intranasal ectopic teeth.

Intranasal ectopic teeth are usually seen in men and can be seen at every age. Yeung and Lee reviewed the literature and found a total of 41 cases. The age of discovery of the intranasal teeth ranged from 3 to 62 years (3). In 1998, Moreano et al. reported a case of an intranasal tooth in a 7- year-old patient (4). In 2001, Lee presented a series of 13 cases, of which seven were children and six were adults. Nine cases were male and four cases were female (5). Our case is the case of a 34-year-old male patient.

The etiology of intranasal teeth is controversial. Many theories have been proposed, including developmental disturbances, such as cleft palate, teeth displaced by trauma, cysts, infection, obstruction to eruption secondary to crowding of dentition, persistent deciduous teeth or dense bone (6).

In most of the reported cases, clinical signs and symptoms are facial pain, external nasal deformities, foul-smelling rhinorrhea, recurrent epistaxis, oronasal fistula, and nasal septal perforation (1-6). In our case, foul-smelling rhinorrhea, left nasal obstruction, and sometimes epistaxis were observed. There was also an ectopic tooth present in the right side of the nasal cavity; but intranasal extension was minimal and covered by intranasal mucosa. The right intranasal ectopic tooth was asymptomatic.

Treatment of intranasal teeth is early extraction, upon diagnosis, because of the potential morbidity. Extraction of intranasal teeth under endoscopic guidance has the advantages of good illumination, clear visualization, and precise dissection (6). In our experience, extraction of nasal teeth under endoscopy guidance is satisfactory. In this case, the symptoms regressed and there were no complications.

## Conclusion

Intranasal ectopic teeth are rarelly seen bilaterally in the nasal cavity. Extraction of intranasal teeth under endoscopic guidance is an adequate treatment. If ectopic intranasal teeth are asymptomatic, clinicians should follow with clinical examination and radiological imaging.
